# Supplementary material for: Trans-(−)-Kusunokinin: A Potential Anticancer Lignan Compound against HER2 in Breast Cancer Cell Lines?
Source: Molecules. 2021 Jul 27;26(15):4537. doi: 10.3390/molecules26154537 (PMC8348432; doi:10.3390/molecules26154537)
Supplement: Supplementary file 1 [file molecules-26-04537-s001.zip › molecules-1278895-supplementary.pdf]

Supplementary Materials

Article

Trans-(−)-Kusunokinin: A Potential Anticancer Lignan Compound against HER2 in Breast Cancer Cell Lines?

Thidarath Rattanaburee [1], Tanotnon Tanawattanasuntorn [1], Tienthong Thongpanchang [2], Varomyalin Tipmanee [1,*] and Potchanapond Graidist [1,*]

[1] Department of Biomedical Sciences and Biomedical Engineering, Faculty of Medicine, Prince of Songkla University, Songkhla 90110, Thailand; thi325@gmail.com (T.R.); thawaii39@gmail.com (T.T.)

[2] Department of Chemistry and Center of Excellence for Innovation in Chemistry (PERCH-CIC), Faculty of Science, Mahidol University, Bangkok 10400, Thailand; tienthong.tho@mahidol.ac.th

* Correspondence: tvaromya@medicine.psu.ac.th (V.T.); gpotchan@medicine.psu.ac.th (P.G.); Tel.: +66-74-45-1743 (V.T.); +66-74-45-1184 (P.G.)

Table S1. PDB identification code of ten breast cancer associated protein and Pubchem CID of selected known inhibitors

Selected protein	PDB ID	Known inhibitor	PubChem CID
Human epidermal growth factor receptor 2 (HER2)	3PP0	03Q<br>Neratinib	16736274<br>9915743
Human epidermal growth factor receptor 1 (HER1)	4WKQ	Gefitinib	123631
Human epidermal growth factor receptor 4 (HER4)	3BBT	Lapatinib	208908
Estrogen receptor (ER)	1SJ0	E4D	448577
Progesterone receptor (PR)	1E3K	R18	71311629
Cyclin-dependent kinases 4 (CDK4)	2W96	Palbociclib	5330286
Cyclin-dependent kinases 6 (CDK6)	5L2I	LQQ	5330286
Poly)ADP-ribose (polymerase (PARP)	5DSY	UHB	72193895
Phosphoinositide-3 kinase (PI3K)	3L54	LXX	46174165
Ras-related protein Ral-A (RalA)	6P0N	NLS	145994356

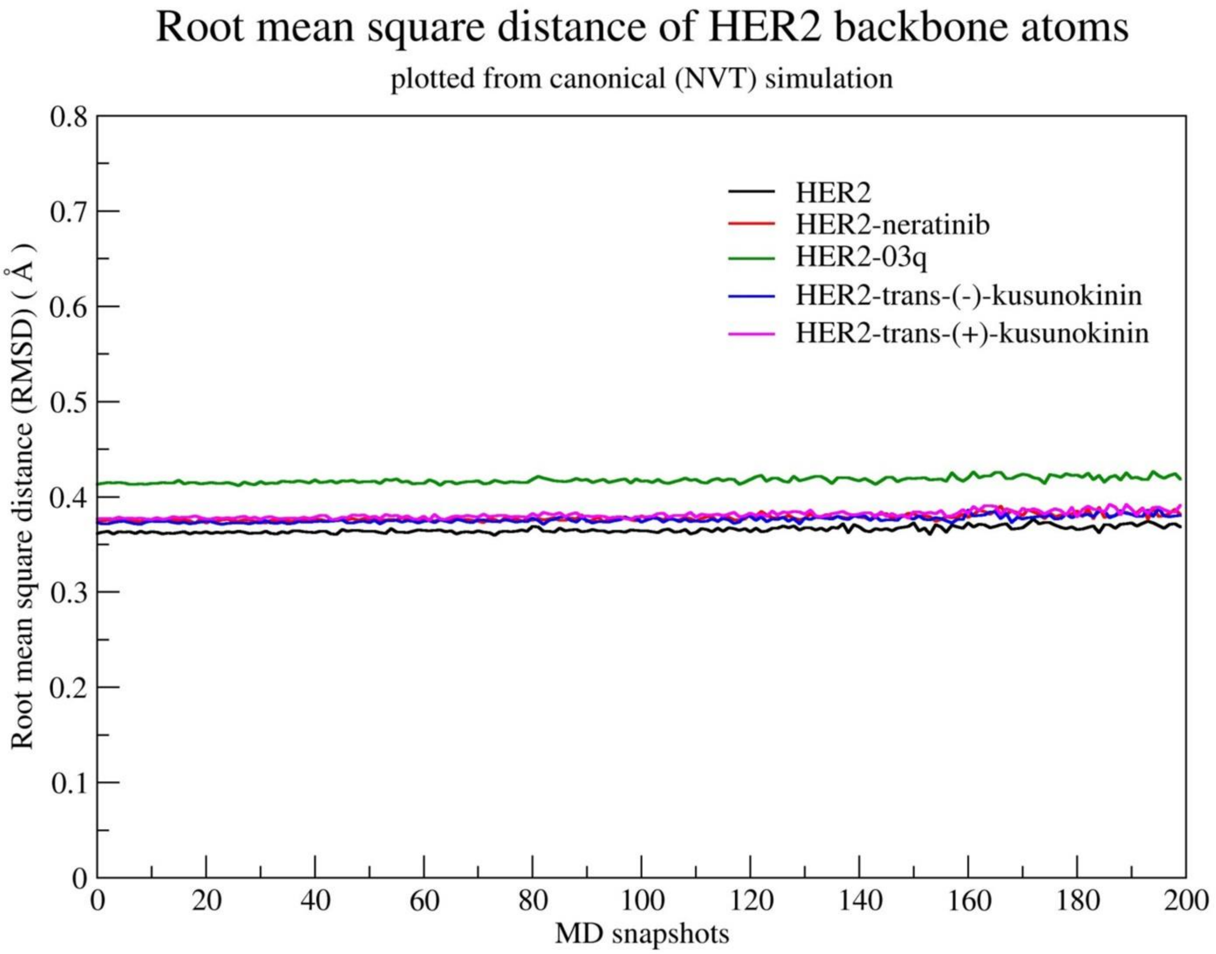

Figure S1. Root mean square distance of HER2 backbone atoms. All MD simulations were carried out under canonical (NVT) ensemble in which all protein atoms of HER2 were restrainted using harmonic potential. The MD simulation consisted of 200 equidistant snapshots from 1000 ps. The root mean square distance was in a unit of angstrom (Å).

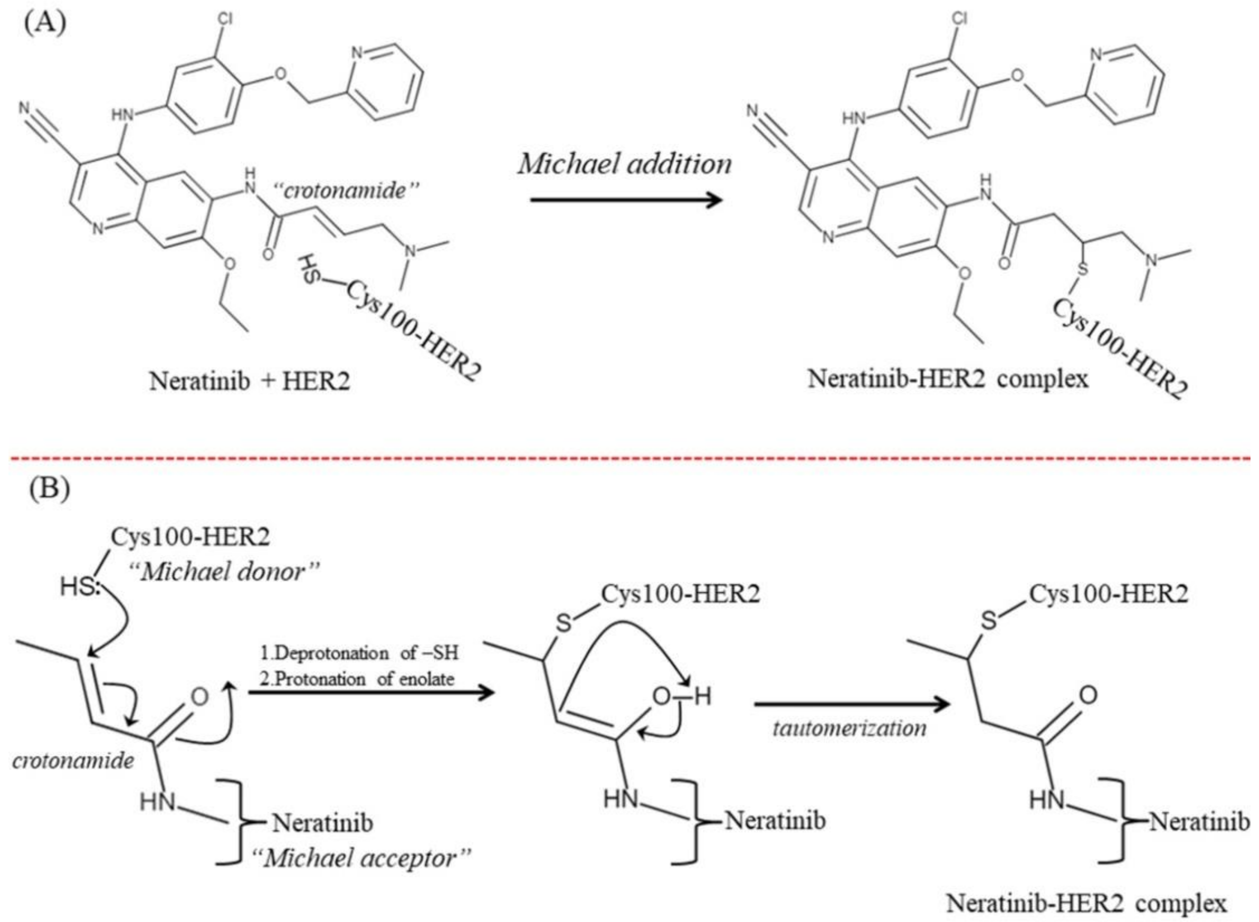

Figure S2. Covalent bond formation between neratinib and Cys100 in ATP binding domain via Michael addition. (**A**) Crotonamide in neratinib formed carbon-sulfur bond with Cys100 of HER2 protein, yielding irreversible binding. (**B**) Reaction mechanism of Michael addition leading to covalent bond formation of neratinib to HER2.
